# The oral-gut-brain axis: how periodontitis influence depression

**DOI:** 10.3389/fmicb.2026.1778744

**Published:** 2026-04-10

**Authors:** Zhixuan Li, Chengzhi Xie, Chunling Pan

**Affiliations:** 1 Liaoning Provincial Key Laboratory of Oral Disease, Department of Periodontics, School and Hospital of Stomatology, China Medical University, Shenyang, Liaoning, China; 2 The First Affiliated Hospital of Dalian Medical University, Dalian, China

**Keywords:** depression, gut microbiota, microbial regulation, oral microbiota, periodontitis

## Abstract

Depression has a high global prevalence and is a common mental–emotional disorder that severely jeopardizes human health. However, current treatment options remain limited, necessitating the exploration of novel pathological mechanisms and intervention targets. Recent studies indicate that periodontitis, as a prevalent chronic oral infectious disease, not only causes local microbial dysbiosis and inflammatory responses but may also influence central nervous system function through the “oral-gut-brain axis,” thereby contributing to the pathogenesis and progression of neurodegenerative diseases such as Alzheimer’s disease, Parkinson’s disease, and multiple sclerosis, as well as neuropsychiatric disorders like depression. This review systematically examines the impact of periodontitis on oral microbiota and its subsequent translocation and colonization in the gut microbiota through pathways including swallowing and bloodstream circulation, ultimately leading to structural and functional dysregulation of the gut microbiota. The interaction between oral and gut microbiota can influence the brain through the “gut-brain axis,” including disturbances in neurotransmitter metabolism, activation of systemic immune responses, and direct or indirect effects of bacterial metabolites (such as short-chain fatty acids, lipopolysaccharides, etc.) on the blood–brain barrier and neural function. This suggests that periodontal health management may serve as a novel strategy for the prevention and treatment of depression. This article further summarizes the potential of oral interventions for periodontitis (such as mechanical debridement and local/systemic antimicrobial therapy), microbiota modulation methods (such as probiotics, prebiotics, and fecal microbiota transplantation), and multidisciplinary collaborative comprehensive treatment strategies in improving microbial homeostasis and alleviating depressive symptoms. Finally, this paper points out the current research limitations in mechanistic details, causal relationships, and clinical translation, while envisioning the feasibility and prospects of developing personalized treatment strategies by targeting the “oral-gut-brain axis” in the future.

## 1 Introduction

The prevalence of mental disorders, such as major depressive disorder (MDD), affects approximately 19% of the general population, significantly impairing patients’ quality of life and work capacity, and potentially leading to suicidal behavior. Depression-related suicide ranks as the tenth leading cause of death in the United States (Park and Zarate, 2019). The pathophysiology of depression remains incompletely understood, with antidepressants based on the monoamine hypothesis currently representing the most common treatment for depressive episodes. However, the therapeutic efficacy of antidepressants varies considerably among individuals and proves ineffective for many patients with major depressive disorder, resulting in frequently unsatisfactory recovery rates (Fox and Lobo, 2019).

Periodontitis is a chronic inflammatory disease primarily caused by infections with anaerobic Gram-negative bacteria. Its main manifestations include alveolar bone resorption and the destruction of periodontal connective tissue, ultimately resulting in tooth loss. Given the close correlation between global population growth, increasing aging, and tooth loss rates, periodontitis presents a significant public health challenge (Kassebaum et al., 2014). Research indicates that the growing prevalence of western ultra-processed foods has an adverse impact on the onset and progression of gingivitis and periodontitis (Discepoli et al., 2026; Cassiano et al., 2022; Coll et al., 2025). In addition to poor dietary habits, highly prevalent chronic conditions such as diabetes and obesity have also been identified as high-risk comorbidities of periodontitis, with a bidirectional pathological relationship existing between them (Chapple and Genco, 2013; Bitencourt et al., 2023). A growing body of research indicates that the detrimental effects of periodontitis extend beyond the oral cavity; its pathogenic bacteria and their metabolites are closely associated with brain disorders, including psychiatric illnesses and neurodegenerative diseases (Hashimoto, 2023). The destruction of periodontal tissues has been shown to be closely linked with subgingival microbiota. An increase in the abundance of subgingival bacteria and specific alterations in its composition can lead to damage of periodontal tissues, while the inflammation caused by periodontitis can, in turn, alter the oral subgingival microbiota, creating a vicious cycle (Schätzle et al., 2003). As the initial segment of the human digestive tract, dysbiotic oral microbiota may colonize the gut and participate in regulating the intestinal ecosystem (Lira-Junior and Boström, 2018). Concurrently, rapid advances in gut-brain axis research have revealed close associations between gut microbiota and neuropsychiatric disorders. Through various signaling pathways—such as neuroendocrine communication, immune modulation, and microbial metabolite production—the gut microbiota modulates central nervous system function, which subsequently affects cognition, emotion, and behavior.

Consequently, periodontitis may influence brain tissue through oral and gut microbiota and their metabolites, thereby mediating the onset and progression of psychiatric disorders. This review aims to explore the mechanisms by which periodontitis promotes depression through the regulation of oral and gut microbiota, as well as potential therapeutic strategies.

## 2 Periodontitis shapes the oral microbiota and metabolite profiles influencing the oral-gut axis

The oral bacteria of periodontitis patients exhibit greater diversity, with up to 10 bacterial genera dominating the condition (Liu et al., 2023). Compared to healthy states, their subgingival microbiota displays dysbiosis, characterised by increased pathogenic bacteria and reduced beneficial bacteria. This shift is influenced by multiple factors, including genetic and environmental influences.

**Table 1:** The effects of smoking, dietary, hormonal and diabetes factors on oral and gut microbiota.

Influencing factors	Site of action	Key research findings (microbiota alterations/mechanisms)	References
Smoking	Oral microbiota	1. Smoking can alter the structure of oral microbiota, characterized by a reduction in commensal bacteria (such as *Neisseria*, *Lautropia*, and *Haemophilus* spp.) and an increase in opportunistic pathogens (such as *Prevotella* spp. and *Streptococcus* spp.), with site specificity. Smoking may lead to changes in the redox state of the oral environment, affecting local inflammatory responses and tissue homeostasis.	Galvin et al. (2023) and Sato et al. (2020a)

		2. Smokers exhibited lower abundances of *Neisseria* and *Capnocytophaga*, but higher abundances of *Streptococcus* and *Megasphaera.* Metagenomic functional prediction further revealed the significant roles of nitrate reduction and tricarboxylic acid cycle-related pathways, suggesting that smoking may also affect microbial metabolic functions. These microbial structures and their functions tended to recover after smoking cessation, indicating that quitting smoking may help regulate abnormalities in the oral microbiota.	Sato et al. (2020b)
		3.16S rRNA gene sequencing revealed that smoking is one of the significant factors influencing oral microbiota variation, second only to familial genetic influence. This finding suggests that oral microorganisms may serve as important mediators in the impact of smoking on disease development and progression, while also providing potential avenues for the prevention and treatment of nasopharyngeal carcinoma.	Liao et al. (2025)
		4. Smoking and grain types have significant effects on the composition of oral microbiota. The increase in *Atopobium* and the decrease in *Neisseria* and family-level *Porphyromonadaceae* serve as hallmark indicators of smoking’s impact on oral microbial composition.	Ryu et al. (2024)
Diet	Oral microbiota	Cereals with higher levels of phenols, lower glycemic index, and higher fiber content (such as barley) are positively associated with the health of oral and gut microbiota.	Ryu et al. (2024) and Wang Y. et al. (2024)
Hormonal factors	Oral microbiota	1. Due to the increased hormones such as estrogen and progesterone during pregnancy, the susceptibility of periodontal tissue to microorganisms increases. The pathogenic groups *Prevotella* and *Atopobium parvulum* were significantly higher in the third trimester than in the pre-pregnancy period.	La et al. (2022)
		2. Through 16S rDNA sequencing, there were differentially distributed genera, among which *Neisseria*, *Porphyromonas*, and *Treponema* were over-represented in the pregnant group, while *Streptococcus* and *Veillonella* were more abundant in the non-pregnant group. In addition, 53 operational taxonomic units were observed to have positive correlations with sex hormones in a redundancy analysis, with *Prevotella* spp. and *Treponema* spp. being most abundant.	Lin et al. (2018)
		3. The microbiota of pregnant women also varies across different body regions. *Lactobacillus* is the most abundant genus in C, V, and U samples (with average proportions of 76, 77, and 59%, respectively), while its average relative abundance is significantly lower in oral (2.8%) and rectal (6.2%) samples.	Kõljalg et al. (2025)
Diabetes	Oral microbiota	1. Even in patients with type 2 diabetes mellitus (T2DM) who have healthy oral conditions, changes occur in their oral microbiota and its metabolites. In the T2DM group, *P. gingivalis* and *Prevotella melanogena* are significantly enriched.	Li et al. (2023)
		2. In diabetic patients, the number of *Fusarium* and *Actinomyces* is significantly higher, while the number of *Proteobacteria* is lower. However, compared with diabetic patients without gingival bleeding, those with both gingival bleeding and diabetes exhibit a marked reduction in *Actinomyces* and an increase in *Bacteroides*. The study results confirm changes in the composition of oral microbiota under different blood glucose levels and at different stages of periodontal disease.	Matsha et al. (2020)
		3. GDM cases showed lower *α*-diversity, with increased *Selenomonas* and *Bifidobacterium* in the oral microbiota, while *Fusarium* and *Leptospira* were reduced.	Xu et al. (2020)
		4. Var*ietans* and *Veillonella* spp. identified in children with type 1 diabetes mellitus (T1D) who exhibit poor glycemic control.	Carelli et al. (2023)
Smoking	Gut microbiota	1. Smoking can directly or indirectly affect the composition and diversity of intestinal microbiota by inducing intestinal oxidative stress, disrupting intestinal barrier function, thereby increasing intestinal permeability, promoting pathogenic bacterial colonization, and triggering inflammatory responses.	Mignini et al. (2024)
		2. Smokers exhibited a significantly increased abundance of *Coprococcus* genus, suggesting that serum *γ*-glutamylcysteine (γ-Glu-Cys) levels may serve as an important mediator in the potential link between smoking-induced gut microbiota alterations and associated diseases.	Kondo et al. (2022)
		3. Maternal smoking during pregnancy alters the gut microbiota structure of offspring infants, leading to increased diversity of Firmicutes phylum and excessive butyrate production, which mediates the elevated risk of overweight and obesity in offspring children. Early breastfeeding can partially mitigate the negative effects caused by smoking.	Peng et al. (2024)
		4. Fermented black barley can significantly alleviate smoking-induced gut microbiota dysbiosis in mice, but its underlying mechanism remains unclear.	Zhong et al. (2022)
		5. P*araprevotella_clara* partially mediates the association between smoking and type 2 diabetes mellitus onset.	Zhang et al. (2025)
		6. Smoking directly reduces the abundance of beneficial bacteria (such as *Intestinimonas* and *Ruminococcaceae*), and can also affect neurotransmitters through the formation of a “positive feedback loop” via metabolites, thereby reinforcing smoking behavior. Additionally, *Actinobacteria* and *Bifidobacterium* may play a critical role in smoking addiction.	Fan et al. (2023)
		7. E-cigarette vapor significantly alleviated the HFD-induced elevation of serum triglyceride and free fatty acid levels, while exacerbating systemic inflammatory responses. However, it showed no significant impact on gut microbial diversity. This suggests that the intervention mechanisms of e-cigarettes and traditional cigarettes on gut microbiota differ, and dietary factors may interact with smoking in complex ways.	Chen et al. (2023)
High-fat diet (HFD)	Gut microbiota	1. A HFD can alter the diversity and composition of the gut microbiota, manifesting as reduced microbial diversity and significant changes in the abundance of specific bacterial populations. HFD rapidly modifies the gut microbiota composition, particularly by increasing the functional capacity of microbiota related to redox reactions, leading to elevated levels of reactive oxygen species (ROS) in the gut and impairing intestinal barrier integrity.	Zeng et al. (2024)
		2. HFD is closely associated with increased abundance of the *Lachnospiraceae*_FCS020 group and decreased abundance of *Bacteroides* and *Barnesiella* genera in the gut microbiota. These microbial alterations show significant correlations with markers of systemic chronic inflammation (SCI), such as elevated IL-2RA levels, suggesting that gut microbiota dysbiosis is a key mechanism in HFD-induced SCI, and IL-2RA represents a potential therapeutic target for SCI.	Du et al. (2025)
Hormonal factors	Gut microbiota	1. The study suggests that the interaction between hormones and gut microbiota may lead to observed gender differences in early childhood. In the age groups of 15–44 and 45–64 years, the incidence of Salmonella infection is significantly higher in females.	Peer et al. (2021)
Diabetes	Gut microbiota	1. The gut microbiota of gestational diabetes mellitus (GDM) cases exhibited significant differences in *β*-diversity, with an increase in gamma-proteobacteria and Haemophilus.	Xu et al. (2020)
		2. The composition of gut microbiota was significantly abnormal in IGT, CGI, and untreated T2D, and was closely associated with insulin resistance. The reduction of butyrate-producing bacteria was a common feature in both prediabetic and end-stage diabetic groups.	Wu et al. (2020)
		3. In common study results, *Bifidobacterium*, *Lactobacillus*, *Fecalibacterium*, *Akkermansia* and *Roseburia* were negatively correlated with T2D, while *Ruminococcus*, *Fusarium* and *Blautia* were positively correlated with T2D.	Gurung et al. (2020)

### 2.1 The effects of periodontitis on pathogenic bacteria and metabolites

Periodontal pathogens aggregate on teeth through dental plaque biofilms, which create a relatively stable habitat for bacteria. A metagenomic study found that periodontitis is closely associated with significant changes in the composition of the subgingival microbiota (Zhao et al., 2025; Han and Ding, 2025). These bacteria directly damage the gingival epithelium and connective tissue via toxins (e.g., lipopolysaccharides, LPS) and gingipains. Concurrently, an immune response leads to the release of inflammatory mediators, resulting in an exaggerated inflammatory response that causes further damage to periodontal structures (Bitencourt et al., 2023). Furthermore, periodontitis alters the oral microbiota by the creating of an acidic and anaerobic microenvironment while disrupting of host immune tolerance. Such an acidic milieu promotes the colonization and proliferation of acid-tolerant and acid-producing periodontal pathogens, such as *Porphyromonas gingivalis*. The proteases released by these bacteria further accelerate the destruction of periodontal tissues, establishing a vicious cycle (Rosier et al., 2024; Moțoc et al., 2023). Moreover, *Fusobacterium nucleatum*, a common commensal and pathogen in oral biofilms, plays a significant role in periodontitis. It is an important producer of ammonia and hydrogen sulide (H2S). As a gaseous signaling molecule, H2S exhibits notable cytotoxicity, capable of inducing host cell apoptosis, impairing mitochondrial function, and promoting local inflammatory responses. Peptidoglycan, a component of bacterial cell walls, though not a bacterial toxin itself, has degradation fragments that act as potent inflammatory activators and play a central role in driving destructive inflammation in periodontal tissues (Tantivitayakul et al., 2020). Simultaneously, the hypoxic conditions within deep periodontal pockets facilitate the proliferation of anaerobic bacteria while inhibiting aerobic and facultative anaerobic species., and consequently drives a shift in the microbial community composition (Wang et al., 2022).

Host genetic factors, such as specific immune-related genes, may indirectly regulate the composition and balance of oral microbiota by influencing immune response states. E−/p-selectin (Niederman et al., 2001) play crucial roles in regulating oral microbiota. In mouse models with E−/p-selectin knockout, alveolar bone resorption is observed, correlating with alterations in the quantity and composition of the oral polymicrobial community (Qi et al., 2021). E−/p-selectin are essential in inflammatory responses; their absence induces immunosuppression, allowing subgingival pathogenic bacteria to proliferate excessively, which results in microbial dysbiosis (Niederman et al., 2001; Hashim et al., 2021). Common environmental factors contributing to oral microbiota dysbiosis include smoking, obesity, stress, medication, oral hygiene practices, and hormonal factors (Asl et al., 2025), etc. Smoking fosters an environment that promotes the proliferation of specific anaerobic pathogens by compromising blood supply and vasculature, impairing fibroblast function, and inducing a state of heightened inflammation, immune suppression, and oxygen depletion (Palmer et al., 2005). These factors collectively alter the oral microbial composition and increase the abundance of pathogenic bacteria (Bizzarro et al., 2013; Hagenfeld et al., 2020). The metabolic dysregulation associated with obesity may influence subgingival microbial composition by elevating levels of periodontal pathogens, potentially increasing the risk of both the initiation and progression of periodontitis (Tamashiro et al., 2023; Xu et al., 2025). Metagenomic studies have revealed that different geographical origins and peri-implant health influence the oral microbiota (Alexandre et al., 2025; Song et al., 2025).

### 2.2 Characteristics of periodontitis-associated oral microbiota

**Figure 1:**
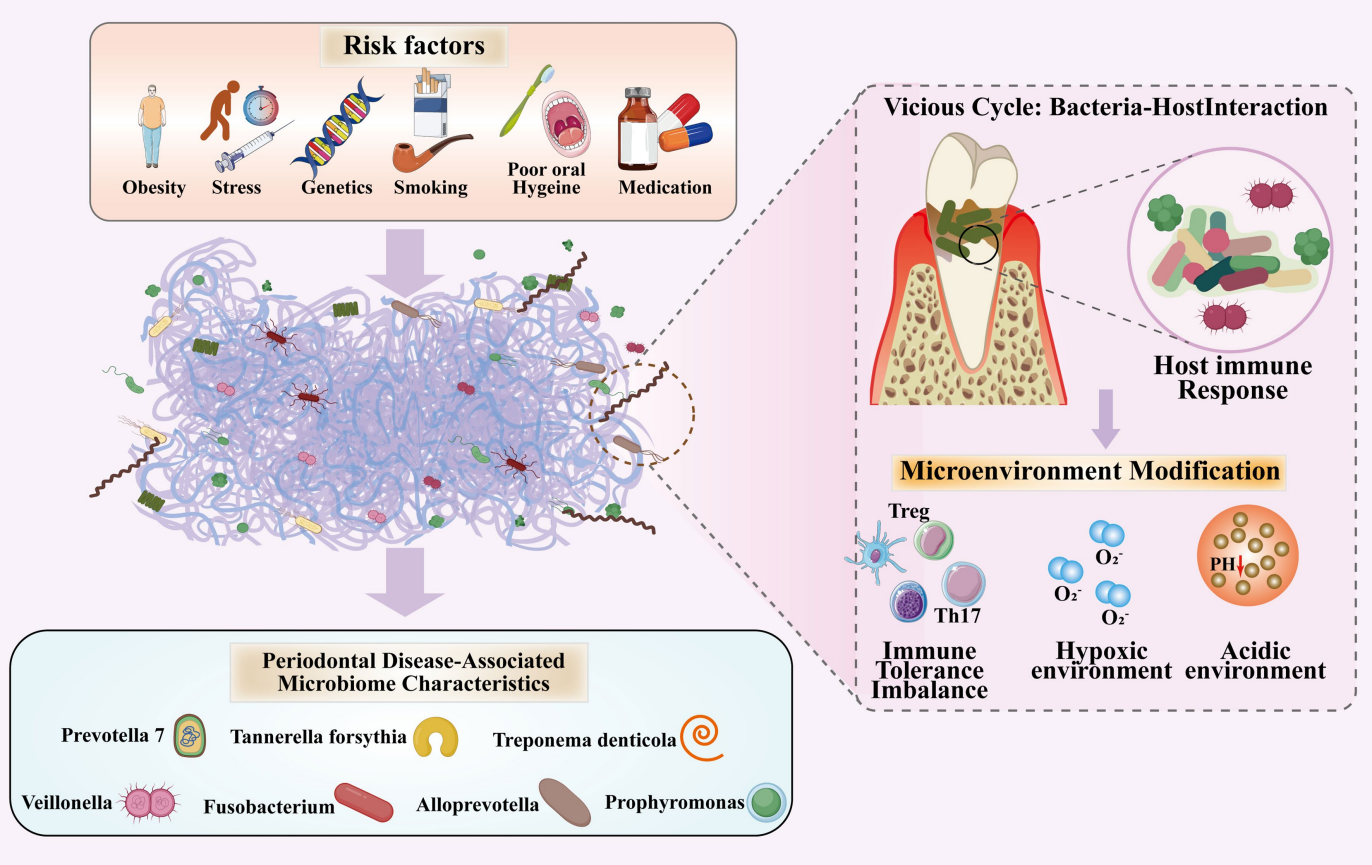
This diagram illustrates the risk factors associated with periodontitis and their interaction with the oral microbiota: the influence of genetic and environmental factors on the oral microbiota (environmental factors include: smoking, obesity, stress, medication, oral hygiene); highlighting interactions with oral microbiota in periodontitis progression: altered microenvironments, dysregulated immune responses, and the vicious cycle of hypoxia and acidification play pivotal roles; outlining periodontitis-associated microbial communities.

Numerous studies have detected genetic material of *P. gingivalis* and *F. nucleatum* in various non-oral pathological tissues, such as DNA of these bacteria has been identified in colorectal cancer tissues, atherosclerotic plaques, and placental tissues (Cui et al., 2025; Ma Z. et al., 2024). The major virulence factor of *P. gingivalis*, LPS, triggers extensive immune-inflammatory responses by binding to Toll-like receptor 4 (TLR4) on host cells, leading to tissue destruction and inducing persistent, low-grade systemic inflammation. This constitutes a critical mechanism by which periodontitis affects systemic health (Figure 1).

## 3 The oral-gut microbiome axis

### 3.1 Possible associations between oral microbiota and gut microbiota

The oral cavity, as the initial segment of the digestive tract, serves as a crucial habitat for microbial communities. Despite anatomical continuity, the oral and intestinal tracts maintain microbial independence through physiological barriers such as gastric acid and bile (Cheung et al., 2023). Nevertheless, these environmental barriers, microbial migration and metabolic interactions occur between oral and intestinal microbiomes: first, through direct entry into the digestive tract through swallowed saliva and food; and second, through the bloodstream when the oral mucosal barrier is compromised, thereby affecting distant organs. Particularly during intestinal dysbiosis, oral bacteria are more readily colonized in the gut. Jeong et al. (2023) conducted 16S rRNA sequencing on saliva and fecal specimens from 112 healthy Koreans, revealing significant associations between oral and intestinal microbial types. Similarly, patients with acute myeloid leukemia (AML) exhibited markedly increased abundance of oral bacteria, such as *Blautia* and *Parabacteroides* within their gut microbiota (Pötgens et al., 2024). These findings suggest that the oral microbiota may influence the structure of the gut microbiota through an intricate network.

The colonization and migration of microbiota within the host not only occupy ecological niches but also activate the host’s immune recognition system, with inflammasomes playing a central role. Bacteria or their components (such as LPS and bacterial DNA) originating from the oral cavity may serve as PAMPs upon migrating to the gut, directly activating inflammasomes in intestinal epithelial cells or immune cells, thereby becoming key ligands for inflammasome activation. Particularly in the context of impaired intestinal barrier function or dysbiosis, overactivated inflammasomes can lead to excessive release of pro-inflammatory factors such as interleukin-1β and widespread pyroptosis of epithelial cells. This intense inflammatory response disrupts the tight junctions between intestinal epithelial cells, significantly increasing intestinal mucosal permeability, resulting in the so-called “leaky gut” state (Nadella and Kanneganti, 2024). Furthermore, the persistent inflammatory environment driven by inflammasomes consumes local oxygen, creating an anaerobic microenvironment in the gut that alters the microbial ecology and may contribute to the development of related diseases (e.g., inflammatory bowel disease, colorectal cancer) (Li et al., 2026). Animal studies have validated these hypotheses: abnormal activation of NLRP3 inflammasomes can drive excessive intestinal inflammatory responses, while functional defects in NLRP3 in intestinal epithelial cells or immune cells may impair the clearance of oral migratory bacteria, exacerbating colitis (Luo et al., 2023). Therefore, the inflammasome pathway serves as one of the critical immunological bridges through which oral microbiota influences the intestinal environment and health, and its precise regulation is essential for maintaining mucosal homeostasis.

### 3.2 Effects of oral microbiota on gut microbiota

#### 3.2.1 Bacterial transfer: how oral bacteria migrate to and colonize the gut

Humans ingest 1–1.5 liters of saliva into the gut daily through swallowing, a process that represents a significant pathway for oral bacteria to disrupt the balance of gut microbiota. Research demonstrates that collecting saliva from patients with periodontitis and transplanting it into mice via oral administration or intragastric gavage reveals abnormal enrichment of oral pathogens in the mouse gut. This confirms that microorganisms from periodontal saliva can enter the intestine and disrupt gut microbiota (Bao et al., 2022). Furthermore, this study identified a specific subtype of *F. nucleatum*, designated FnaC2, derived from the oral pathogen associated with periodontitis, which exhibits high adaptability to the acidic gastrointestinal environment. This finding was initially suggested by 16S rRNA sequencing analysis of the *F. nucleatum* microbiota collected from patients with colorectal cancer and non-cancer controls. Subsequent validation through animal experiments, in which mice were gavaged with this bacterial subtype, confirmed its enhanced survival and activity within the gastrointestinal tract (Zepeda-Rivera et al., 2024). These studies further substantiate the migration pathway through which oral microbiota influence gut microbiota via swallowing, offering novel therapeutic strategies for refractory gut-microbiota-associated systemic diseases. However, differences in oral and gut microbiota between humans and mice may confound experimental outcomes. Consequently, subsequent research requires optimized animal models or human clinical trials to refine these mechanisms.

Moreover, studies indicate that oral bacteria can enter the bloodstream through routine brushing or dental procedures, triggering transient bacteremia and subsequently circulating to the gut (Lockhart et al., 2008). In patients with severe periodontitis, subgingival plaque may enter the bloodstream due to increased mucosal permeability caused by inflammation, subsequently affecting distant organs and inducing chronic inflammation. This process is closely associated with the development of inflammatory diseases such as coronary atherosclerosis, inflammatory bowel disease (IBD) (Miyauchi et al., 2025), neurodegenerative disorders (Qian J. et al., 2023; Qian X. et al., 2023) and colorectal cancer (Xiao et al., 2021; Zhou et al., 2025; Kwong et al., 2018). Concurrently, periodontal bacteria such as *F. nucleatum* and *P. gingivalis* can colonize dendritic cells or macrophages and damage extraoral tissues (Xue et al., 2018). A retrospective analysis of individual blood samples revealed that individuals with *F. nucleatum* or streptococcal bacteremia exhibited heightened susceptibility to colorectal cancer (Kwong et al., 2018). Furthermore, *F. nucleatum* in feces and blood showed a positive correlation among colorectal cancer patients, with a combined blood microbiome and blood microbiome fragmentomic profile aiding in the early detection of colorectal cancer, thereby alleviating the economic burden on patients (Zhou et al., 2025). For the clinical diagnosis of colorectal cancer, plasma testing confers greater diagnostic sensitivity compared to fecal testing. Furthermore, most circulating bacterial DNA fragments are predominantly derived from the gastrointestinal and oral microbiota (Xiao et al., 2021). This suggests a future therapeutic strategy involving the design of specific antibiotics coupled with probiotic administration, aiming to precisely inhibit periodontal pathogens and modulate microbial ecology, thereby offering a potential avenue for treating systemic diseases associated with dysbiosis.

#### 3.2.2 Regulation of gut microbiota by oral microbiota metabolites

Metabolites play a crucial role as mediators in the interactions between oral and gut microbiota. High concentrations of short-chain fatty acids (SCFAs) can be detected in the supernatants of cultured periodontal pathogenic bacteria (e.g., *P. gingivalis*). *P. gingivalis* can produce SCFAs through proteolysis, and the concentration of SCFAs in the gingival crevicular fluid (GCF) of periodontitis patients is positively correlated with periodontal clinical indicators (probing depth, bleeding index, attachment loss). The concentration of volatile sulfur compounds (VSCs, with H₂S as the main component) in the oral cavity of periodontitis patients is significantly elevated and correlates with the severity of periodontal inflammation. The periodontal pocket is a blind sac with a depth of several millimeters, and its anaerobic environment facilitates the local accumulation of gas H₂S. High concentrations of H₂S in the oral cavity can damage periodontal tissues, leading to the entry of bacteria and their products into the bloodstream through ulcerated gingival vessels, thereby reaching the intestines. Additionally, the large amounts of inflammatory factors produced can trigger systemic inflammation, affecting the gut microbiota (Dahlstrand Rudin et al., 2021; Ratcliff and Johnson, 1999).

Specifically, metabolites produced by oral bacteria, such as *P. gingivalis*, including SCFAs and H₂S, can reach the gut through the bloodstream or via swallowing. These metabolites directly inhibit the growth of beneficial gut bacteria while promoting the proliferation of opportunistic pathogens. Moreover, they can compromise the integrity of the intestinal epithelial barrier, increase permeability, and activate intestinal immune cells to secrete pro-inflammatory factors, leading to dysbiosis of the gut microbiota (Kunath et al., 2024).

### 3.3 Reverse regulation of oral microbiota by gut microbiota

#### 3.3.1 Reverse regulation of oral microbiota by gut microbiota via the immune system and endocrine system

Systemic immune responses modulated by the gut microbiota further influence the local oral immune environment through immune cell migration and signaling pathways, thereby indirectly regulating the composition and function of the oral microbiota (An et al., 2025). Following gut immune activation, activated immune cells and effector molecules such as cytokines [e.g., Interleukin-17 (IL-17), Tumor Necrosis Factor-alpha (TNF-*α*)] can promote local inflammatory responses, alter the oral microecological environment, and either inhibit or promote the growth of specific microbial communities, playing a pivotal role in the regulation of oral microbiota (Nagao et al., 2022). SCFAs, neurotransmitters, and hormonal substances produced by gut microbiota through dietary fiber metabolism exert effects not only locally within the gut but also enter the bloodstream, influencing energy balance and inflammatory states, thus indirectly altering the structure and function of oral microbiota. In patients with insomnia, dysbiosis of both gut and oral microbiota is accompanied by alterations in multiple serum metabolite levels, providing a molecular basis for understanding the complex interactions between gut-oral microbiota and the endocrine system (Lin et al., 2024). Analyzing compositional and functional shifts in gut microbiota demonstrates significant potential as a target for the early diagnosis and treatment of oral diseases such as periodontitis.

#### 3.3.2 The effects of smoking, dietary, hormonal and diabetes factors on oral and gut microbiota

Table 1 summarizes the factors influencing the oral and gut microbiota, including smoking, diet, and other related factors.

## 4 The relationship between periodontitis, microbiota and depression

### 4.1 Clinical correlation between periodontitis and depression

The association between periodontitis and depression is supported by a growing body of evidence synthesized in systematic reviews and meta-analyses.

A cross-sectional analysis within the NHANCE study examined the association between the severity of periodontal disease (PD) and intrinsic capacity (IC) in 551 elderly participants. The findings revealed a strong correlation between severe periodontitis and impaired mental health within IC manifested as depressive symptoms. This suggests that improving periodontal health in older adults may contribute to maintaining their psychological wellbeing (Dibello et al., 2025). Concurrently, cross-sectional studies demonstrate a significant correlation between periodontitis and anxiety levels (Liang et al., 2025; Xu et al., 2025; Kolte et al., 2025). Psychological stress and related disorders (PSRD) negatively impact the outcomes of periodontal treatment outcomes (Mauland and Neupane, 2025), resulting in inadequate improvement in clinical attachment loss (CAL) and periodontal pocket depth (PDD). Furthermore, anxiety and depression frequently co-occur, thereby increasing the disease burden and the risk of suicide. A study combining cross-sectional and prospective cohort designs found that periodontal disease is associated with an increased risk of depression, anxiety, and their comorbidity. Specifically, individuals with periodontal disease exhibited a 26.5% higher risk of developing comorbid depression and anxiety compared to those without periodontal disease (Wang J. et al., 2024). This study first revealed the longitudinal association between periodontitis and the comorbidity of depression and anxiety, addressing the limitations of previous research that was predominantly confined to cross-sectional designs, and offering new perspectives for the prevention of depression and anxiety.

However, current genetic methodologies have yet to provide robust evidence for a causal relationship between the two (Pi et al., 2024). A univariable Mendelian randomization analysis identified a causal relationship between the risk of periodontal disease and increased anxiety and stress-related disorders (ASRD). Nonetheless, this association diminished when confounding factors, such as smoking behavior and educational attainment, were included in the multivariate analysis (Hu et al., 2024). Therefore, more sophisticated and comprehensive genetic approaches are necessary to investigate this relationship in greater depth.

### 4.2 Oral microbiota and depression

#### 4.2.1 Relationship between oral microbiota dysbiosis and depression, post-traumatic stress disorder (PTSD)

Significant differences exist in the composition and metabolic profiles between of oral microbiota individuals exhibiting depressive symptoms and healthy controls (Lou et al., 2024). During episodes of depression, anxiety, and PTSD, the *β*-diversity of oral microbiota is markedly reduced (Qiu et al., 2025). A large-scale 16S rRNA sequencing study identified *Prevotella histicola* as being associated with PTSD and depression, while Eggerthia was correlated with poor psychotherapeutic responses (Malan-Muller et al., 2024). In animal studies, the transplantation of salivary microbiota from chronic restraint stress (CRS) mice into germ-free (GF) mice induced dysbiosis, such as Pseudomonas enrichment, and led to depressive-like behavior in the GF recipients (Lou et al., 2024). Furthermore, IgG positivity for *P. gingivalis* serotype K1 correlates with moderate-to-severe depressive symptoms during early pregnancy (Postolache et al., 2025), thereby providing novel insights into the connection between periodontal disease and mental health.

#### 4.2.2 Potential mechanisms linking oral microbiota to depression

Table 2 summarizes key experimental findings regarding the mechanisms through which oral microbiota may influence neuropsychiatric disorders.

**Table 2:** Exploring the mechanisms linking oral microbiota and depression.

Mode of action	Key mechanism	Specific mechanism of action/evidence	Key experimental findings/functional implications	References
Immune pathways	Infection with *P. gingivalis* promotes central nervous system inflammation by activating peripheral ZAP70/NF-κB pathways to drive Th1 cell differentiation.	*In vivo* experiments utilised combined techniques including flow cytometry, haematoxylin and eosin staining, and Evans blue dye leakage to assess inflammation, demyelination, BBB permeability, and peripheral blood Th1 cell ratios in EAE mice. *In vitro* experiments validated that *P. gingivalis*-LPS stimulates Th1 differentiation via the ZAP70/NF-κB pathway.	*P. gingivalis* infection significantly elevated the proportion of Th1 cells in the peripheral blood of EAE mice. *P. gingivalis* infection exacerbated inflammation and demyelination within the central nervous system of EAE mice, while also increasing blood–brain barrier permeability. *P. gingivalis*-LPS stimulated Th1 differentiation pathways; employing pathway inhibitors reduced both Th1 cells and pro-inflammatory factors.	Dai et al. (2025)
	Periodontitis promotes M1 polarisation of microglia through Stat3-mediated Th17 imbalance	Establishment of a Stat3 knockout mouse model in Th17 cells (cKO-LPS group) to observe the proportion of Th17 cells and M1 microglia in the brain	The imbalance of Th17/Treg cells in peripheral immune organs (the spleen) and the central nervous system (the brain) was improved, and M1 polarisation of microglia within the brain was reversed.	Zhou et al. (2025)
	Periodontal disease-associated oral microbial dysbiosis impairs splenic immune responses in diabetic mice	Saliva samples from healthy volunteers and periodontitis patients were transplanted into the oral cavities of diabetic mice, whereupon inflammatory markers in the mice’s periodontal tissues were assessed.	Salivary microbiome from periodontitis patients Significantly increased expression levels of TNF-*α* and IL-1β in periodontal tissues of mice, alongside upregulation of Th17 cell abundance	He et al. (2022)
Neural pathway	Dysbiosis of the oral microbiota influences neuropsychiatric disorders through tryptophan metabolism.	16S rRNA sequencing analysis report examining the structure and composition of the salivary microbiome in the psychological health symptom group (n = 306) and the psychological health control group (n = 164).	Dysbiosis of the oral microbiota leads to reduced plasma 5-HT levels, which may be associated with inhibition of the tryptophan metabolic pathway.	Malan-Muller et al. (2024)
	Dysbiosis of the oral microbiota influences neurotransmitters and depressive behavior via the gut-brain axis.	Transplanting oral saliva from CRS model mice into GF mice to observe gut microbiota composition and neurotransmitter levels	CRS-induced oral dysbiosis can disrupt the oral and intestinal barrier function in GF mice (via decreased link protein expression), increase BBB permeability, and lead to neurotransmitter imbalances (such as 5-HT), thereby triggering depressive-like behavior.	Lou et al. (2024)
	*P. gingivalis* can activate microglia.	Using immunohistochemical analysis, the degree of microglial activation in the hippocampus and prefrontal cortex of obese mice inoculated with *P. gingivalis* via the gingiva was examined.	Gingival application of *P. gingivalis* increases microglial cell body size in the hippocampus and prefrontal cortex of obese mice, alongside microglial activation.	Oue et al. (2024)
	Periodontal pathogen *P. gingivalis* and its metabolites induce neuroinflammation	Establish a bacteremia model to observe its effects on neuroinflammation in the brain	Chronic inflammatory states induced by oral pathogens (such as *P. gingivalis*) may further exacerbate neuroinflammation by compromising the BBB, facilitating the entry of inflammatory mediators and bacterial metabolites into the brain. This compromises neuronal function and survival, thereby establishing a vicious cycle.	Lei et al. (2023)
	Bacterial exosomes can migrate to the brain via the vagus nerve and bloodstream, thereby inducing neuroinflammation and depression-like behaviors in mice.	esEVs are transported to the hippocampus via the vagus nerve, and their levels decrease in the hippocampus after vagus nerve transection.	Gastric instillation of esEV mimics bacterial effects, inducing depression-anxiety-like behaviors and neuroinflammation, while vagotomy reduces the transport of esEV to the hippocampus.	Ma X. et al. (2024) and Dabkeviciute and Kriauciunas (2025)
Endocrine pathway	The oral microbiota may participate in the pathological processes of psychiatric disorders by influencing the HPA axis and associated hormone levels.	Resting-state functional magnetic resonance imaging (rsfMRI) scans were conducted on 139 subjects, and their oral microbiota were assessed.	Significant alterations in the oral microbiota structure have been identified in individuals with schizophrenia, potentially linked to abnormal brain functional connectivity via the HPA axis.	Lin et al. (2024)
	Oral microbiota influence brain function via endocrine pathways	Transplanting oral saliva from CRS model mice into GF mice to observe changes in hormone levels and brain function	Changes in the oral microbiota accompany alterations in hormone levels and behavioural abnormalities; docosapentaenoic acid alleviates these symptoms.	Xu et al. (2025)
	The regulation of neuroendocrine factors by the oral microbiota holds significant functional importance.	Observing the effects of oral probiotics on the peptidome of different brain regions	Oral probiotic intervention modulates the brain’s polypeptide profile, influencing the expression of neurotrophic factors including BDNF, thereby alleviating stress-induced hyperactivation of the HPA axis.	Zhang et al. (2020)

### 4.3 Gut microbiota and depression

#### 4.3.1 Role of gut dysbiosis in depression

During depressive episodes, microbial richness and diversity consistently decrease (Zhernakova et al., 2016; Liu et al., 2024a; Radjabzadeh et al., 2022). An animal study on gut microbial composition revealed significant *β*-diversity differences between chronic unpredictable mild stress (CUMS) rats and control rats (Meng et al., 2025), which aligns with observational findings (McGuinness et al., 2022). A large-scale cross-sectional study (N = 7,656) utilizing metagenomic sequencing indicated that depressive disorders [including MDD, dysthymic disorder, and any depressive disorder (AnyDep)] were associated with 17 bacterial species (e.g., reduced *Ruminococcus bromii,* increased *Eggerthella lenta*), whereas anxiety disorders [including generalized anxiety disorder (GAD) and any anxiety disorder (AnyAnx)] were linked to 3 bacterial species (e.g., reduced *Bifidobacterium bifidum* and *Coprococcus eutactus*). These associations were independent of psychotropic drug (PTD) effects, revealing distinct links between internalizing disorders and the gut microbiome (Brushett et al., 2023). Using the same methodology, gut microbial diversity was found to correlate closely with the severity of MDD. Cross-sectional studies employing fecal metagenomic sequencing demonstrated significantly reduced *α*-diversity in the gut microbiota of patients with moderate to severe depressive disorder compared to controls. Furthermore, *Bacteroides* abundance was markedly elevated in patients with moderate to severe depressive disorder, while *Ruminococcus* and *Eubacterium* levels were reduced in the severe group. The study identified 99 bacterial species specifically associated with depression severity and constructed 37 microbial markers capable of distinguishing patients based on different levels of depressive disorder severity (Hu et al., 2023). An analysis of 16S rRNA sequencing from fecal samples collected from 268 participants over a 2-year follow-up period revealed that a reduced relative abundance of *B. bifidum* correlated with poorer current cognitive function. Furthermore, future cognitive decline was associated with a reduced abundance of the *Firmicutes phylum* and *Intestinibacter*, while worsening depressive symptoms were linked to a decreased abundance of the *Bacteroidetes phylum.* These findings suggest that the composition of the gut microbiome may predict the trajectory of cognitive function and depressive symptoms in later life (Kolobaric et al., 2024).

## 5 Integrated mechanisms linking periodontitis, microbiota, and depression

Gut dysbiosis is recognized as a significant risk factor for depression, potentially influencing brain function and behavioral expression by altering immune system activation, neurotransmitter synthesis, and the production of metabolic byproducts.

### 5.1 Immune-inflammatory pathways

As the largest immune organ in the human body, the gut harbors 70% of immune cells and coordinates immune function through microbial metabolites such as SCAFs, thereby regulating neuroinflammatory responses (Mann et al., 2024). Emerging evidence indicates that the gut microbiota plays a critical role in the pathogenesis of depression, often in close association with the translocation of oral microbiota, particularly periodontal pathogens. Oral microorganisms associated with periodontitis can enter the digestive tract, alter gut microbial composition, and subsequently promote depression-related behaviors through immune pathways. The gut microbiota regulates depressive-like behaviors by activating Th17 cells. Patients with depression and learned helplessness mouse models exhibit increased abundances of Th17-inducing bacteria, notably *Clostridium symbiosum*. Transplantation of these bacteria induces social deficits, while Th17 cell deficiency abolishes these effects (Medina-Rodriguez et al., 2023). In the context of periodontitis, oral pathogens such as *P. gingivalis* may exacerbate depressive-like behaviors by promoting Th17 cell differentiation. DSS-induced colonic inflammation promotes Th17 differentiation and induces depressive-like behaviors, while modulation of the Th17/Treg balance reverses this phenomenon (Xia et al., 2023). Invasion by oral microbiota can amplify inflammatory responses. Pro-inflammatory factors in the murine gut can trigger neuroinflammation (Lan et al., 2025), and Morganella species participate in the pathogenesis of depression by activating TLR2/TLR1 receptors to induce IL-6 (Bang et al., 2025). In depression models, pro-inflammatory Bacteroides levels are elevated, while anti-inflammatory Clostridium and *Bifidobacterium longum* CECT 30763 are reduced (Liu et al., 2024b; Tamayo et al., 2025; Xu et al., 2024). Colitis also modulates gut microbiota via TLR-4, stimulating microglia-mediated neuroinflammation and inducing anxiety-like behaviors (Wang et al., 2025). Dysbiosis, such as reduced abundance of *Lactobacillus murinus*, promotes anxiety progression by amplifying neuroinflammation (Weng et al., 2024). Microglia, as the primary immune effector cells in the brain, are regulated by the gut microbiota and can induce neuroinflammation while impairing hippocampal neurogenesis, thereby mediating depressive and anxiety disorders (Agirman and Hsiao, 2021). The gut microbiota modulates microglial phenotypic transformation, consequently triggering depressive-like behaviors (Huang et al., 2023). In summary, periodontitis-associated oral microbiota may contribute to the pathogenesis and progression of depression by modulating gut microbiota composition and engaging immune-inflammatory pathways involving Th17 cell differentiation and microglia-mediated neuroinflammation.

### 5.2 Neurotransmitter and neuroendocrine regulation

Periodontitis-related oral dysbiosis can disrupt the gut microbiota via multiple pathways, contributing to depression through neurotransmitter and neuroendocrine mechanisms. Gut microbiota directly influence mood regulation by synthesizing or modulating neurotransmitters such as 5-hydroxytryptamine (5-HT) and gamma-aminobutyric acid (GABA). Animal studies utilizing the CUMS model have validated the influence of 5-HT-mediated gut microbiota on depressive pathways. It was found that *Lactobacillus lactis* E001-B-8 increased hippocampal 5-HT levels in CUMS mice, ameliorated depressive-like behavior, and modulated gut microbiota composition, for example, by enhancing the abundance of *Lactobacillus* (Ma et al., 2023). Similarly, interventions such as fluoxetine or metformin restore gut microbial diversity, increase beneficial bacteria (e.g., *Akkermansia muciniphila*), and raise 5-HT levels (Xie et al., 2025; Lee et al., 2025). On the other hand, GABA, as the primary inhibitory neurotransmitter, exhibits production closely linked to gut microbiota (Radjabzadeh et al., 2022). The research indicates that heightened activity in GABA degradation pathways correlates with more severe depressive symptoms (Kolobaric et al., 2024). Metagenomic analyses have revealed compositional differences in the gut microbiota of patients with MDD, bipolar disorder (BD), and schizophrenia (SZ), particularly marked by an increased abundance of GABA-metabolizing bacteria such as *Eggerthella* (McGuinness et al., 2022). In a mouse model of postpartum depression (PPD), GABA levels correlate with specific taxa, including *Mucispirillum schaedleri* and *Bifidobacterium pseudolongum*. Moreover, 919 syrup reverses depressive-like behavior while modulating GABA and microbiota (Tian et al., 2021). Additionally, gut microbiota influence neurotransmitters via metabolites and vagal pathways. For instance, CRS induced reductions in *Parabacteroides* and *Ruminococcus*, correlating with lower monoamine levels (Yang et al., 2021). Conversely, fecal microbiota transplantation from stressed mice can alter neurotransmitter profiles in recipients via vagal activation (Siopi et al., 2023). Periodontitis-induced oral dysbiosis can exacerbate the axis by activating the HPA axis and increasing cortisol. This disruption affects brain function via endocrine and neural pathways, worsening neuropsychiatric symptoms like anxiety and depression (Breivik et al., 2014). Abnormal intestinal immune signals reach the brain via the vagus nerve and blood, driving neurological symptoms like depression (Qian J. et al., 2023; Ke et al., 2023). Concurrently, these signals also activate central immune cells and regulate neurotransmitter metabolism, influencing brain inflammation and the “gut-brain” axis balance (Shin et al., 2023). This “oral-gut-brain” axis represents a key pathological pathway in depression.

### 5.3 Metabolic and barrier dysfunction

The oral microbiota dysbiosis associated with periodontitis disrupts the intestinal microenvironment through metabolic regulation and impaired barrier function, contributing to the pathogenesis of depression. Gut microbiota exert broad regulatory effects on host metabolism, producing key metabolites such as SCFAs and tryptophan derivatives that maintain blood–brain barrier (BBB) integrity, modulate neuroinflammation, and support brain energy metabolism. Melatonin has been shown to alleviate depressive behavior by inhibiting *Alistipes inops* and improving systemic tryptophan metabolism (Zheng et al., 2024). *Hypericum perforatum* redirects the kynurenine pathway toward tryptophan metabolism, reducing neuroinflammation and ameliorating depressive-like behavior in chronic restraint stress mice (Liu et al., 2023). Oral SCFA supplementation ameliorates anxiety-like behavior and alters gut microbiota composition in CUMS rats (Behrens et al., 2024). Metabolomic analyses have revealed lipoprotein abnormalities associated with *Clostridium* and *Proteus* species in major depressive disorder (Amin et al., 2023). Periodontitis increases BBB permeability through peripheral and central inflammatory responses (Bitencourt et al., 2023; Jiang et al., 2025b; Martin-Hernandez et al., 2023). Peripheral inflammatory mediators may also activate the vagus nerve to transmit signals to the central nervous system (Capuron and Miller, 2011). Additionally, periodontitis-induced HPA axis activation elevates cortisol levels, exacerbating gut microbiota dysbiosis and impairing microbial metabolite production. This increases intestinal permeability, enabling gut-derived inflammatory factors to enter systemic circulation and sustain chronic inflammation (Luo et al., 2025; Ravenda et al., 2025). Together, these mechanisms establish a pathological axis linking oral dysbiosis to intestinal metabolic disturbance and central nervous system dysfunction in depression.

### 5.4 Microbial translocation and direct neural effects

**Figure 2:**
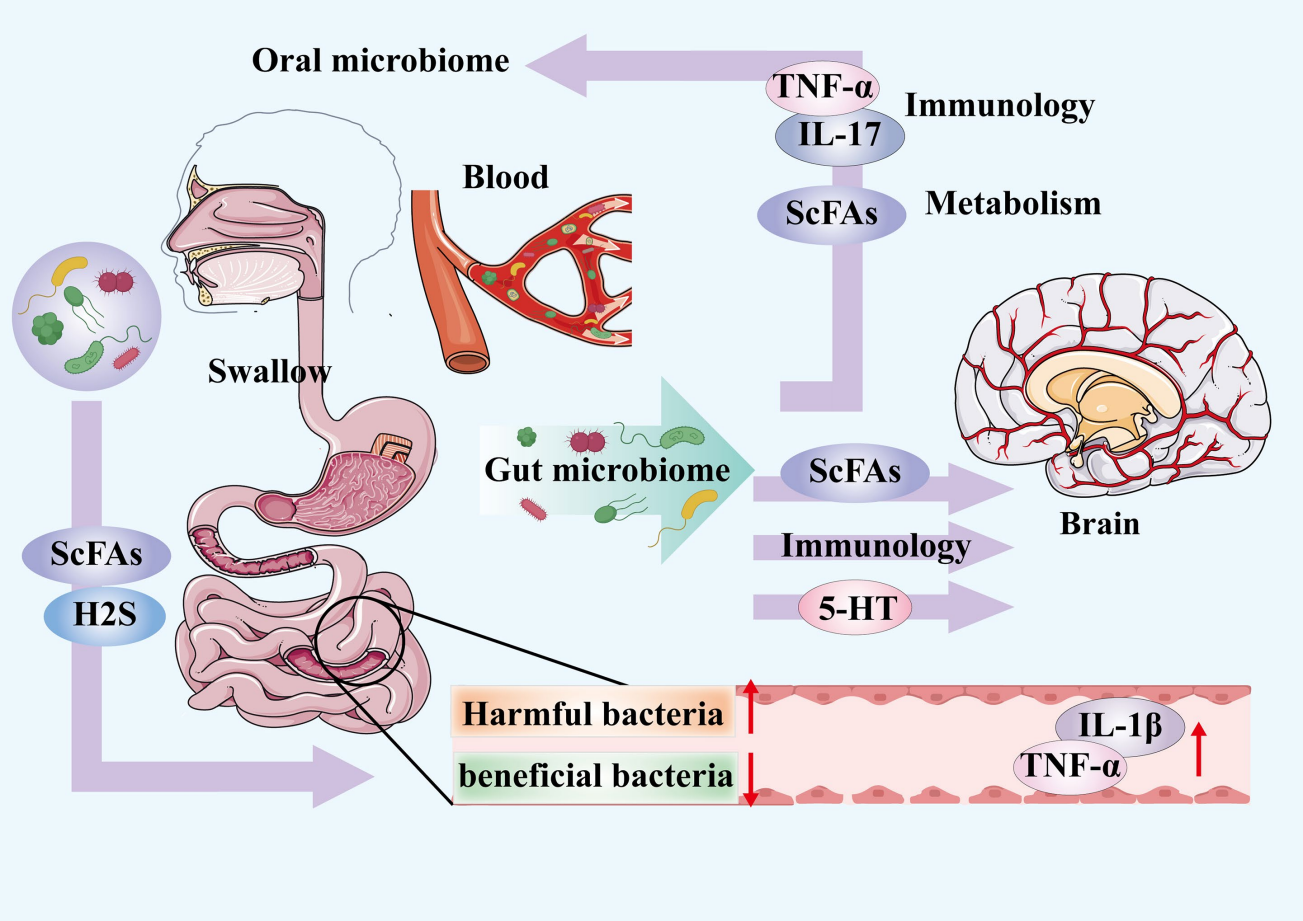
This is a schematic diagram illustrating the ‘oral-gut-brain’ axis. The oral-gut axis is introduced: oral microbiota colonise and influence gut microbiota via two pathways—ingestion and the bloodstream. Furthermore, gut microbiota can reciprocally regulate oral microbiota through metabolic byproducts; the gut-brain axis is introduced: gut microbiota influence brain function by affecting the production of metabolic byproducts and neurotransmitters, as well as by activating the immune system.

Multiple studies have confirmed that periodontitis-associated oral microbiota can ectopically colonize the gut, representing a key pathological pathway linking periodontitis to depression. 16S rRNA sequencing of healthy Korean populations first identified oral-origin taxa such as Streptococcus and Haemophilus in the gut microbiome (Jeong et al., 2023), and a subsequent study detected 24 ectopically colonized oral species in the gut (Cha et al., 2025). In a case of odontogenic brain abscess, both pus culture and metagenomic sequencing revealed elevated abundances of *P. gingivalis* and Streptococcus stellatus (Sun et al., 2025), suggesting that oral microbes may further migrate to the central nervous system and participate in neuroinflammatory processes. The gut microbiota plays a critical role in neuroinflammation via the “gut–brain” axis, a bidirectional network involving neural, endocrine, immune, and microbial pathways. Fecal 16S rRNA sequencing of patients with PTSD identified periodontitis-associated genera—including Mitsuokella, Odoribacter, Catenibacterium, and Olsenella—that correlate with PTSD severity (Malan-Muller et al., 2022). In DSS-induced mouse models, oral administration of periodontitis-derived salivary microbiota (PSM) exacerbated anxiety-like behaviors, altered gut microbiota composition, reduced cortical neuron counts, and activated microglia; these effects were abolished by antibiotic intervention (Qian J. et al., 2023). In summary, periodontitis-associated oral microbiota colonize the gut via ectopic migration and contribute to neuroinflammation, neuronal dysfunction, and glial activation through microbial translocation and neural pathways, thereby influencing the development of depression and anxiety. These findings underscore oral microbes as upstream regulators of the gut–brain axis and provide a microbial–neural perspective on the pathological link between periodontitis and depression (Figure 2).

## 6 Stabilizing the oral-gut-brain axis

### 6.1 Oral hygiene interventions

Poor oral hygiene is a risk factor for depression. Maintaining good oral hygiene not only helps improve and sustain periodontal tissue health but also contributes to the prevention of depressive episodes. Long-term cohort studies indicate that good oral hygiene habits correlate with a reduced prevalence of periodontitis (Skudutyte-Rysstad et al., 2007). Good oral hygiene reduces the excessive proliferation of pathogenic bacteria and maintains the dominant position of symbiotic bacteria. However, oral hygiene care should maintain a moderate balance to avoid excessive cleaning. Using mouthwash more than twice a day significantly reduces the diversity of the oral microbiota, leading to an increase in *Candida albicans* and *F. nucleatum*. Oral bacteria can influence the diversity of gut microbiota through the “oral-gut” axis. Maintaining good oral hygiene practices is expected to become an emerging target for the prevention and adjunctive management of depression by preserving microecological homeostasis and suppressing chronic inflammation (Adil et al., 2025). Consequently, oral hygiene instruction should prioritize educating patients on systematic approaches to cleaning hard-to-reach areas rather than emphasising specific brushing durations. An analysis of 13 clinical trials, encompassing 607 patients, demonstrated that interdental brushes outperform dental floss in reducing plaque and bleeding indices while exhibiting higher patient acceptance. Thus, interdental brushes constitute the preferred tool for interdental cleaning in periodontitis patients, particularly during maintenance phases (Sanz et al., 2020).

### 6.2 Microbiota modulation

#### 6.2.1 Application of probiotics and prebiotics

Probiotics inhibit the growth of pathogenic bacteria through competitive exclusion mechanisms and also produce antimicrobial peptides and organic acids that modulate local pH, creating an unfavorable environment for pathogens (Gao et al., 2022). Predominantly comprising genera such as Lactobacillus and Bifidobacterium, they play a critical role in maintaining oral and gut microbial homeostasis, regulating microbial balance, and alleviating inflammation. In periodontitis models, probiotic administration has been shown to reduce alveolar bone resorption and ameliorate oral and gut microbiota dysbiosis (Cataruci et al., 2024). qRT-PCR analysis confirmed that the Lactobacillus SMFM2016-NK strain decreased pro-inflammatory cytokine levels in both gingival and colonic tissues of periodontitis-induced rats. Metagenomic sequencing has further demonstrated that fermented dairy products improve the oral and gut microecological environment (Choi et al., 2021). Prebiotics are indigestible substances that serve as substrates for probiotics, promoting the growth and metabolic activity of beneficial bacteria. Fermentation in the gut yields SCFAs, which lower intestinal pH, inhibit harmful bacteria, and enhance intestinal barrier function and immunomodulation (Lin et al., 2021; Socała et al., 2021). Prebiotics promote the proliferation of beneficial bacteria, suppress the growth of periodontal pathogens, maintain oral microecological balance, and alleviate local inflammation. Studies indicate that supplementation with prebiotics such as inulin or *β*-glucan increases the abundance of beneficial bacterial populations, enhances microbial diversity, reduces inflammatory cytokine expression, and facilitates periodontal tissue repair (Colamarino et al., 2023). Clinical trials have confirmed that specific probiotics alleviate symptoms of depression and anxiety, with meta-analyses demonstrating that probiotic interventions reduce both depressive and anxiety symptoms (Asad et al., 2025). Furthermore, combined interventions with probiotics and prebiotics exhibit superior therapeutic efficacy compared to monotherapy, restoring oral and gut microbial balance, enhancing anti-inflammatory effects, and improving neuropsychiatric symptoms (Kezer et al., 2025).

#### 6.2.2 Fecal microbiota transplantation

Fecal microbiota transplantation (FMT) involves the transfer of microbial communities from healthy donors into the intestinal tract of patients to rapidly restore gut microbial balance. In recent years, this therapeutic approach has shown promising applications in the field of neuropsychiatry. Studies have demonstrated that rats subjected to CUMS exhibit improved behavioral outcomes and normalized gut microbiota composition following FMT. These changes are accompanied by increased hippocampal levels of neurotransmitters and neurotrophic factors—including 5-HT, GABA, and brain-derived neurotrophic factor (BDNF)—along with reduced inflammatory markers such as interleukin-6 (IL-6) (Cai et al., 2022). FMT has also shown potential efficacy in patients with treatment-resistant depression, possibly by modulating gut microbiota and their metabolites, thereby influencing host gene expression and immune responses to enhance antidepressant effects (Jiang et al., 2025a). However, current clinical evidence remains preliminary, with a lack of large-scale randomized controlled trials. The long-term safety and efficacy of FMT require further validation. In summary, FMT exerts potential antidepressant effects by modulating gut microbiota to influence neurotransmitter metabolism and immune-inflammatory responses. Nevertheless, its mechanisms of action remain to be elucidated, and clinical application necessitates standardization.

### 6.3 Modulation of oral and gut microbiota composition by diet

The Mediterranean diet, rich in dietary fiber and polyphenols, provides key substrates for microbial fermentation and ecological balance. In the gut, these nutrients selectively promote the growth of SCFA-producing bacteria, including Faecalibacterium and Roseburia. The resulting increase in SCFAs enhances intestinal barrier function and suppresses local and systemic inflammatory responses. Concurrently, dietary polyphenols in the oral cavity inhibit the proliferation of pro-inflammatory pathogens such as *P. gingivalis*, thereby reducing the overall oral microbial load and mitigating potential peripheral sources of inflammation. This bifocal regulatory mechanism extends to the gut, where polyphenols promote the growth of *A. muciniphila*, a keystone species for maintaining mucus layer integrity, while inhibiting opportunistic pathogens (Picchianti Diamanti et al., 2020). The synergistic effects of these microbial shifts, further potentiated by the anti-inflammatory properties of omega-3 fatty acids, suggest that the Mediterranean diet fosters a holistic anti-inflammatory microbial environment (Nikdasti et al., 2025). This ecological remodeling of the “oral-gut” axis presents a plausible pathway through which dietary patterns influence brain function, supporting the emerging hypothesis that the Mediterranean diet exerts antidepressant effects via modulation of inflammatory and neuroendocrine pathways.

### 6.4 Periodontal treatment methods

Periodontal therapy extends beyond local inflammation control, with growing recognition of its systemic effects. Non-surgical periodontal therapy (NSPT) reduces periodontal pathogen load by removing supra- and subgingival biofilms, leading to beneficial remodeling of the gut microbiota—oral-derived opportunistic pathogens decrease in the gut, and microbial communities in both niches shift toward a healthier profile (Baima et al., 2024). This bidirectional modulation of the “oral-gut” axis provides a microecological basis for understanding how periodontal therapy reduces systemic low-grade inflammation. In complex cases, periodontal surgery further stabilizes the oral microecology by reconstructing anatomical structures (Graziani et al., 2017). Post-surgery, oral pathogen levels decrease while probiotic abundance increases (Ho et al., 2025), accompanied by reduced local inflammation and decreased systemic inflammatory burden. At the mechanistic level, ferroptosis and cuproptosis contribute to periodontitis pathogenesis. Periodontal pathogen-induced dysregulation of copper and iron homeostasis promotes tissue destruction through oxidative stress, autophagy, and glutathione pathways (Zheng et al., 2025). Targeting ferroptosis (e.g., regulating SNCA and FTH1 expression) and cuproptosis-related pathways represents emerging therapeutic strategies (Torres et al., 2024; Chen et al., 2024). Integrating these findings into mental health research, periodontal therapy-induced microbiota remodeling, reduced inflammatory burden, and modulation of cell death pathways correspond to key mechanisms in depression—namely, gut microbial dysbiosis, chronic inflammation, and impaired neuronal plasticity. Thus, periodontal therapy may exert adjunctive antidepressant effects via the “oral-gut-brain” axis. This interdisciplinary perspective expands periodontal medicine and offers microecology-based insights for comprehensive depression management.

### 6.5 Integrated treatment strategies

#### 6.5.1 Multidisciplinary collaboration

Advancements in technologies such as 16S rRNA sequencing and metagenomic sequencing have progressively revealed specific alterations in the gut and oral microbiota of depression patients. Metabolomics further elucidates changes in relevant metabolic pathways and metabolites, providing crucial data for understanding how oral and gut microbes influence brain function and facilitating the translation of basic research into clinical applications (Tian et al., 2025). Research indicates that probiotic strains, by improving gut microbiota, can increase the expression of BDNF in the brain, thereby ameliorating depressive-like behavior (Zhang et al., 2023). Integrating findings from multidisciplinary research in neuroimaging, immunology, and microbiomics enables a deeper elucidation of the biological mechanisms of the microbe-brain axis, laying the foundation for the development of multi-targeted therapeutic strategies (Tian et al., 2025). The profound integration of oral medicine, neuroscience, and microbiology has significantly broadened our understanding of neuropsychiatric disorders. As a vital microecosystem within the human body, the bidirectional regulatory mechanisms between the oral microbiota and the nervous system provide new theoretical foundations and potential intervention targets for disease pathogenesis and progression.

#### 6.5.2 Personalized treatment

**Figure 3:**
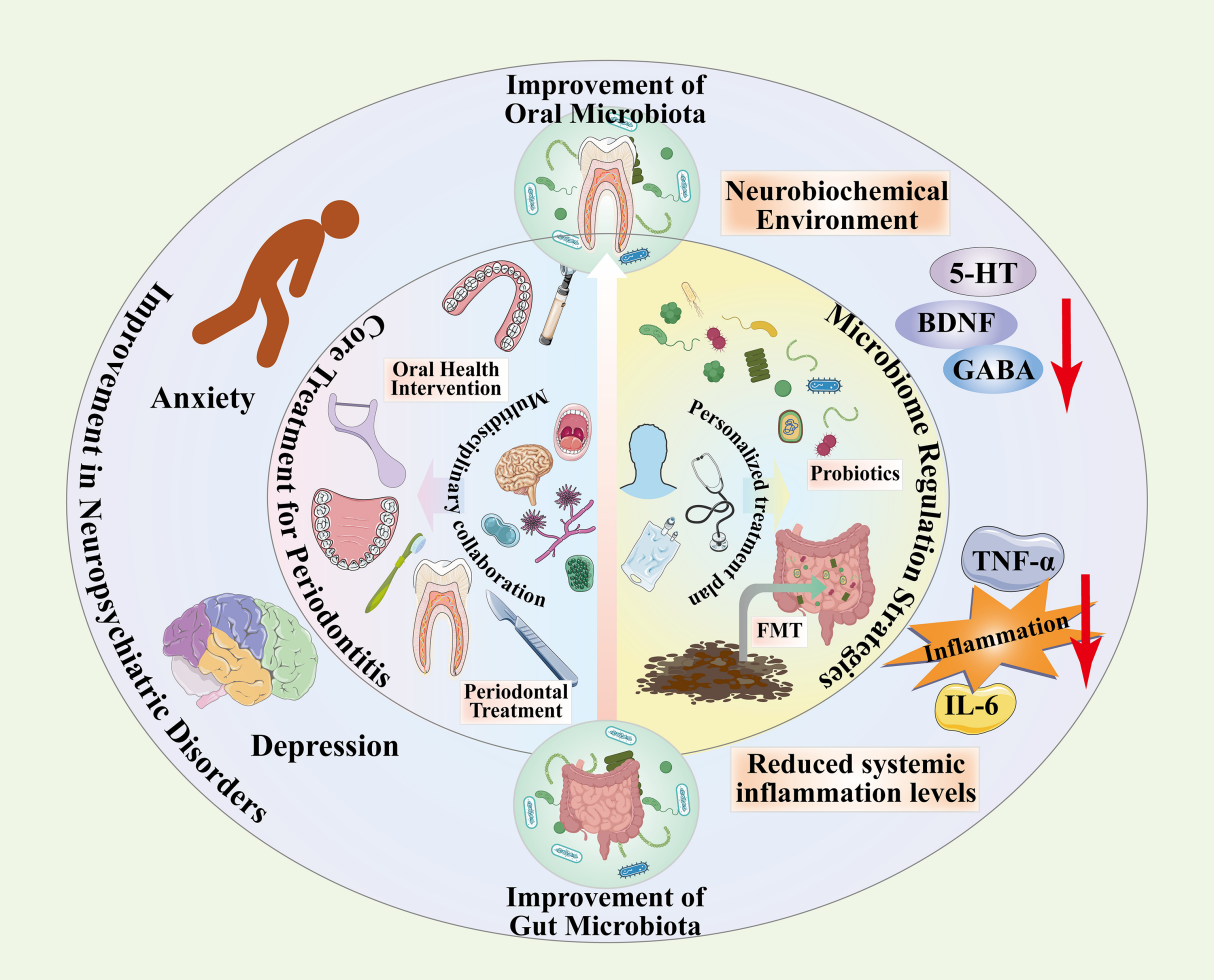
This diagram illustrates the positive impact of improving oral and gut microbiota on neuropsychiatric disorders such as anxiety and depression. It outlines the role of oral hygiene guidance, initial periodontal treatment, and surgical intervention in modifying the oral microbiota and their potential implications for neuropsychiatric disorders; it details the regulation of oral and gut microbiota through probiotics, prebiotics, and fecal microbiota transplantation, exploring their mechanisms of action and therapeutic potential for neuropsychiatric conditions; it emphasises the importance of multidisciplinary collaboration and personalised treatment approaches.

Personalized dietary plans are also crucial, as dietary components can significantly influence microbiota composition. Studies have found that high-fat diets (HFDs) can induce dysbiosis in the gut microbiota of mice, subsequently triggering depression-like behaviors through the microbiota-gut-brain axis. Dietary interventions, such as supplementation with specific flavonoids (e.g., ligustrazine II), can ameliorate metabolic disorders and alleviate depressive symptoms by reshaping the gut microbiota, increasing SCFAs production, and reducing systemic and neuroinflammatory responses (Luo et al., 2025). This suggests that collaboration between psychiatry and nutrition departments in developing personalized dietary regimens holds fundamental significance for regulating microbiota at the source and improving depression. Comprehensive intervention plans can be tailored based on individual microbiota profiling results. Additionally, probiotic supplementation, such as *Lactobacillus plantarum* GOLDGUT-HNU082, can significantly alleviate depression-like behaviors induced by CUMS in mice. The mechanism involves reshaping the gut microbiota (e.g., promoting Bifidobacteria growth), restoring key neurotransmitter (e.g., serotonin) balance, reducing inflammatory cytokine (e.g., TNF-*α*) levels, and promoting hippocampal neurogenesis. Future efforts should involve microbiologists and clinicians working together to screen specific strains and intervention regimens with well-defined neuroregulatory functions to achieve precision-targeted microbial therapy (Li et al., 2025). The formulation of personalized treatment plans must comprehensively consider the severity of periodontitis, microbial characteristics, and the specific type of neuropsychiatric disorder, necessitating a thorough risk assessment. The severity of periodontitis is evaluated through clinical indicators such as periodontal pocket depth and the gingival bleeding index, while also incorporating the diversity and degree of dysbiosis within the oral microbiota to inform treatment decisions. Microbiome analysis encompasses not only oral flora but also gut microbiota influences, as both interact through the oral-gut-brain axis to modulate neuropsychiatric disease pathogenesis (Hashimoto, 2023). Therapeutic strategies should integrate pharmacological interventions (e.g., anti-inflammatory and psychiatric medications), oral hygiene management (e.g., regular scaling and enhanced oral cleansing), psychological interventions, and microbiota modulation (e.g., probiotic administration) to create a comprehensive treatment model (Qian J. et al., 2023) (Figure 3).

## 7 Limitations of current research and future research directions

### 7.1 Causal inference issues

The aforementioned studies exhibit several limitations. Firstly, the issue of causal strength arises; cross-sectional studies suffer from ambiguity in causal direction, making it challenging to determine whether dysbiosis is a cause or a consequence of mental illness. The bidirectional regulatory mechanisms between these factors necessitate further longitudinal investigation. Inconsistent Mendelian results and the disappearance of associations following multivariate adjustments suggest that potential confounding factors, such as smoking, educational attainment, and alcohol consumption, may have undermined the genetic evidence supporting causality.

### 7.2 Methodological constraints

Methodological limitations are evident: animal models (e.g., CUMS, DSS colitis) differ from human diseases, and microbiota transplantation studies remain largely restricted to murine models. The efficacy and safety of human microbiota interventions require validation through large-scale clinical trials. Additionally, some studies involve small sample sizes (e.g., 139 schizophrenia patients) or specific populations (e.g., pregnant women, elderly individuals), which necessitates caution when generalizing conclusions.

### 7.3 Unexplored mechanisms and biases

Multiple mechanisms acting synergistically remain unexplored due to insufficient integrated analysis of cross-interactions between immune, neurotransmitter, and metabolic pathways, as well as a lack of systems biology-based models. Finally, the article does not address reverse causality (e.g., the impact of psychotropic drugs on microbiota) or systematically evaluate publication bias, where positive results are more likely to be published.

### 7.4 Clinical translation gaps

Currently, there is a lack of studies demonstrating that periodontal/oral hygiene treatment can improve depressive symptoms, suggesting that the clinical translational potential of oral microecological interventions in mental health remains to be explored. There is an urgent need to design prospective cohort studies or randomized controlled trials to verify the ameliorative effect of periodontal treatment on depressive symptoms and its mechanistic association with gut microbiota and inflammatory markers.

### 7.5 Strengthening causal inference

Future research should employ prospective cohort studies combined with multi-omics integration, including metagenomics, metabolomics, single-cell transcriptomics, and brain imaging, to strengthen causal inference. Mechanistic studies should incorporate intervention trials, such as probiotic or antibiotic interventions, to validate hypotheses.

### 7.6 Deepening mechanistic understanding

The effects of the microbiome may be modulated by host genetic factors, dietary habits, environmental influences, and other individual differences. Future work should aim to establish subtypes of mental disorders based on microbiome characteristics to enable personalized interventions. Additionally, the use of organoids and gene editing technologies is recommended to elucidate the mechanisms by which microbial metabolites, such as SCFAs, penetrate the blood–brain barrier and their specific action targets on neurons.

### 7.7 Advancing clinical translation and expanding research horizons

Future research should focus on identifying oral or gut microbial biomarkers for the early detection of high-risk individuals for neuroinflammation or psychiatric disorders among patients with periodontitis, thereby enabling targeted health management. Concurrently, enhancing research on the relationship between oral health and systemic diseases, particularly neuropsychiatric disorders, will contribute to the advancement of interdisciplinary diagnostic and therapeutic models, facilitating a clinical paradigm shift from localized treatment to holistic health maintenance. Furthermore, it is essential to elucidate the specific pathways of cross-organ migration of oral microbiota, their colonization conditions, and the regulatory details of their influence on “gut-brain” axis signaling, including immune factors, metabolites, and neurotransmitters.

## 8 Conclusions and outlook

Periodontitis promotes the onset and progression of neuropsychiatric disorders, such as depression and anxiety, by regulating oral and gut microbiota, with the “mouth-gut-brain” axis serving as the primary pathogenic pathway. The dysbiosis induced by periodontitis disrupts gut microbiota through the “mouth-gut” axis, while neurotransmitter regulation, immune inflammatory responses, and metabolic effects influence conditions like depression via the “gut-brain” axis. Future research should focus on identifying key oral and gut microbiota, deepening our understanding of the pathogenic mechanisms involved, and developing more effective treatment strategies. Enhanced multidisciplinary collaboration is essential, including the advocacy for the inclusion of oral screening in neuropsychiatric disease prevention guidelines. Intervening in periodontitis and modulating microbiota to treat neuropsychiatric disorders represents a paradigm shift in medicine—from controlling localized infections to addressing systemic diseases. As scientific exploration advances, we may witness the realization of a simple yet revolutionary concept: safeguarding periodontal health is tantamount to protecting our brains.
